# Creation and validation of the harmonized Arabic version of the Expanded Prostate Cancer Index Composite for Clinical Practice (EPIC-CP)

**DOI:** 10.1080/2090598X.2021.2002636

**Published:** 2021-11-28

**Authors:** Mohannad A. Awad, Luke Hallgarth, Ghassan A. Barayan, Mohammed Shahait, Ramiz Abu-Hijlih, Ala’a Farkouh, Raed A. Azhar, Musab M. Alghamdi, Ahmad Bugis, Said Yaiesh, Saad Aldousari, Alaeddin Barham, Mohamed Saed, Ayman Moussa, Waleed Hassen, Shelly Naud, Mark K. Plante, Richard Grunert

**Affiliations:** a Department of Surgery, Division of Urology, University of Vermont Medical Center, Burlington, VT, USA; b Department of Surgery, King Abdulaziz University, Rabigh, Saudi Arabia; c Department of Surgery, Umm Al-Qura University, Makkah, Saudi Arabia; d Department of Surgery, King Hussein Cancer Center, Amman, Jordan; e Department of Surgery, Division of Urology, King Abdulaziz University, Jeddah, Saudi Arabia; f Urology Unit, Department of Surgery, Mubarak Al-Kabeer Hospital, Kuwait, Kuwait; g Department of Surgery (Urology Division), Faculty of Medicine, Kuwait University, Kuwait, Kuwait; h Department of Urology, MD Anderson Cancer Center, Houston, TX, USA; i Urology, Surgical Subspecialties Institute, Cleveland Clinic, Abu Dhabi, United Arab Emirates; j Cleveland Clinic Lerner College of Medicine of Case Western University, Cleveland, OH, USA; k Medical Biostatistics, Larner College of Medicine, University of Vermont, Burlington, VT, USA

**Keywords:** Prostate cancer, quality of life, validation, translation, questionnaire

## Abstract

**Objectives:**

Tocreate and validate a translated Arabic version of the Expanded Prostate Cancer Index Composite for Clinical Practice (EPIC-CP), a validated patient-reported outcome (PRO) widely used for assessing the quality of life in patients with prostate cancer (PCa).

**Patients and Methods:**

Using the established protocol as defined by the Professional Society for the Health Economics and Outcomes Research (ISPOR) for translating patient care questionnaires, a harmonised translated Arabic version of EPIC-CP was created. The questionnaire was tested in native Arabic speakers from four different Arabic countries (Saudi Arabia, United Arab Emirates, Jordan, and Kuwait). Cronbach’s alpha and interclass coefficient correlation (ICC) analyses were used to test the internal consistency and test–retest reliability, respectively. In addition, PCa characteristics were collected for participants.

**Results:**

In total, 168 patients with PCa participated in the study (39 from Saudi Arabia, 23 from United Arab Emirates, 65 from Jordan, and 41 from Kuwait). In all, 52 (31%) participants repeated the questionnaire for test–retest reliability analysis. The median (interquartile range [IQR]) age of patients included in the study was 66 (61–71) years. The median (IQR) PSA level was 9.8 (6–19) ng/mL. Most patients had Grade Group 2 PCa at diagnosis (31%), clinical stage cT1 (42%), managed primarily by urology (79%), and the primary treatment was radical prostatectomy (71%). The total Cronbach’s alpha coefficient was 0.84 demonstrating an acceptable internal consistency. The total ICC was also acceptable at 0.64.

**Conclusion:**

The Arabic version of the EPIC-CP is a reliable and valid tool for assessing health-related quality of life for Arabic patients with PCa.

## Introduction

Prostate Cancer (PCa) treatment options including radical prostatectomy, brachytherapy, and surveillance all have their respective side-effects that vary in severity yet continue to impact health-related quality of life (HRQL) [1]. The combination of prevalence and increased survival rates have led to the need for accurate measuring of HRQL through patient-reported outcomes (PROs) to assist physicians and educate patients in expected treatment outcomes.

To address this need, the Expanded Prostate Index Composite (EPIC) was created as a validated PRO measure tool for HRQL before and after PCa treatment [2]. Based on the University of California Los Angeles-Prostate Cancer Index (UCLA-PCI), EPIC was created in English as a 50-question self-reported questionnaire [3]. The length of this has since been altered into both a 26-question version (EPIC-26) and a 16-question short version, Expanded Prostate Cancer Index Composite-Clinical Practice (EPIC-CP) to create a more concise tool that would be less daunting to patients [4,5]. The EPIC-CP fits onto a single page and the vast majority (96%) of patients are able to complete the questionnaire in <10 min, thereby possibly making this version the most practical for clinical usage [6]. The EPIC-CP covers five domains with 16 items: urinary incontinence (three items), urinary irritation/obstruction (three items), intestinal symptoms (three items), sexual function (three items) and vitality/hormonal symptoms (three items). Similar to EPIC-26, one more separate item measured overall urinary bother not included in any of the domains due to its overlapping effect on both urinary irritation/obstruction and incontinence [4]. When tested against the EPIC-50 and EPIC-26 questionnaires, there is a significant correlation (*r* ≥ 0.93) for all domains as well as high internal validity (Cronbach’s α = 0.64–0.84) when tested internally [6].

The EPIC-26 has been translated and validated into many languages [6–9]. However, the new shorter EPIC-CP version is currently being used in North America and has proven to be practical in routine clinical care of patients with PCa [10]. At present, the EPIC-CP has been translated into two languages (Chinese and Portuguese) and validated to maintain integrity across languages [6,11]. Arabic is the official language in 26 countries and is the fifth most common language spoken with >400 million speakers worldwide [12]. Recent data suggest that PCa incidence is increasing in the Arab world [13]. Saudi Arabia, one of the largest Arabic-speaking countries, has witnessed a profound increase in PCa incidence, with an eightfold change between 1990 and 2016 [14]. Despite this, there remains a need for a validated Arabic version to accommodate a significant portion of the global population. In addition, available data remain scarce about PCa in our region and one of the most important tools to advance high-quality research is to utilise PROs [15]. The aim of the present study was to translate and validate an Arabic version of the EPIC-CP in native Arabic speaking countries.

## Patients and methods

### Translation and cultural adaptation

**Figure 1.**
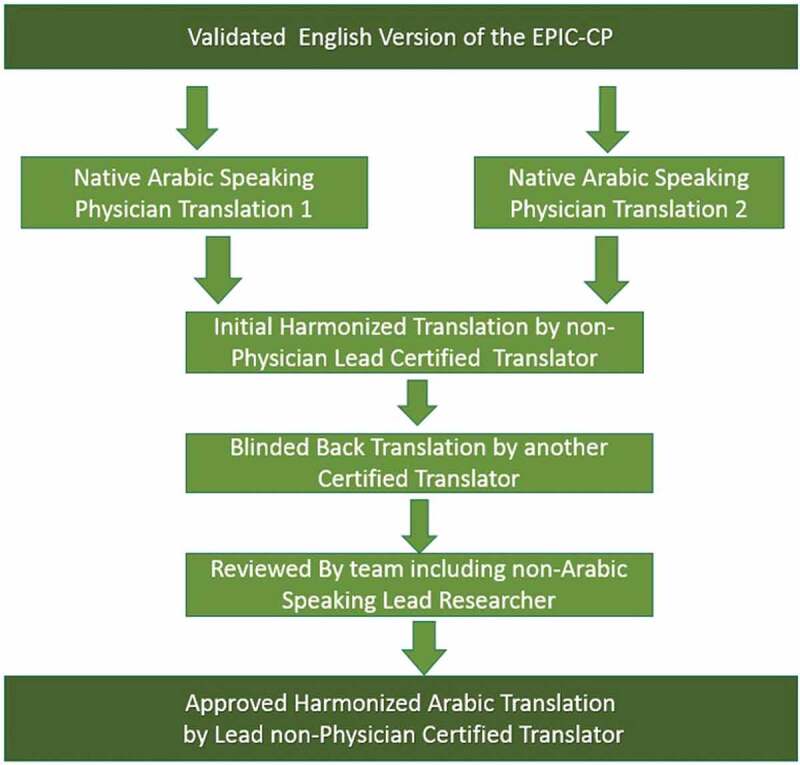
Translation process of EPIC-CP into Arabic.

Using the established protocol as defined by the International Society for Pharmacoeconomics and Outcomes Research (ISPOR) for translating patient-care questionnaires [16], two native Arabic speaking physicians (M.A. and G.B.) separately translated an independent draft of the EPIC-CP into Arabic. This was then reviewed by our non-physician certified lead Arabic translator to create a harmonised Arabic version. In turn, an additional non-physician certified translator then back translated the harmonised version into English. The entire team then collectively reviewed and made changes to this English version to adjust for any cross-cultural differences. The non-physician lead translator then re-translated this finalised version into Arabic to create the final harmonized translation for clinical use with approval by the entire team (Figure 1). The translated Arabic EPIC-CP questionnaire can be seen in Supplement 1.

### Participants and procedure

Four academic institutions from four different Arabic countries collaborated in recruiting Arabic speaking patients with PCa and testing our translated EPIC-CP. In addition to EPIC-CP questions, patients’ and disease characteristics were abstracted from each patient medical record including (age, PSA, group grade, clinical stage, clinical setting, primary treatment, date of primary treatment, adjuvant radiation, salvage radiation, and/or hormonal therapy [yes/no]). The translated EPIC-CP was distributed by E-mail to known patients with PCa through a REDCap hyperlink. The questionnaires were self-administered by patients who agreed to participate in the study. Institutional Review Board approval was obtained from all institutions involved in the study.

### Statistical analysis

Subscale and total scores were scored as described in the original EPIC-CP paper by Chang et al. [5]. Internal consistency of the translated EPIC-CP scores was assessed by Cronbach’s α coefficients. Adequate internal consistency was defined *a priori* as >0.7. For test–retest reliability, in a subsample of participants the EPIC-CP was completed on two different occasions separated by 2–4 weeks. The test–retest reliability was assessed by interclass correlation coefficients (ICCs). An adequate ICC was defined *a priori* as >0.5. The ICCs were based on two-way random effects models using the absolute agreement definition [17]. The ICC reflects the scale’s ability to differentiate among respondents. Standard errors of measurement, reflecting the precision over multiple administrations within a respondent, are the square root of the mean square of error of two-way ANOVA models [17]. When there was no more than one missing item for a subscale, the missing item was set equal to the mean of the available items and the items summed. Analyses were performed using the Statistical Analysis System (SAS) version 9.4 (SAS Institute Inc., Cary, NC, USA) except for the ICCs that were calculated using the Statistical Package for the Social Sciences (SPSS) version 25 (IBM Corp., Armonk, NY, USA).

## Results

In total, 168 patients with PCa participated in the study (39 from Saudi Arabia, 23 from the United Arab Emirates, 65 from Jordan, and 41 from Kuwait). In all, 52 (31%) participants repeated the questionnaire for test–retest reliability analysis. The median (interquartile range [IQR]) age of the patients was 66 (61–71) years. The median (IQR) PSA level was 9.8 (6–19) ng/mL. Most patients had Gleason Grade Group 2 at diagnosis (31%), clinical stage cT1 (42%), managed primarily by urology (79%), and primary treatment was radical prostatectomy (71%). Table 1 summarises the clinical characteristics of the participants.

**Table 1.** Clinical characteristics of participants withPCa

Characteristics	Value
Age, years, median (IQR),	66 (61–71)
PSA level, ng/mL, median (IQR)	9.8 (6–19)
Group Grade*, *n* (%)	
1 (Gleason score <6)	33 (20.9)
2 (Gleason score 3 + 4 = 7)	49 (31)
3 (Gleason score 4 + 3 = 7)	33 (20.9)
4 (Gleason score = 8)	31 (19.6)
5 (Gleason score = 9–10)	12 (7.6)
Clinical T-Stage*, *n* (%)	
cTX	26 (16.5)
cT1	67 (42.4)
cT2	39 (24.7)
cT3–T4	26 (16.5)
Clinical setting, *n* (%)	
Urology	133 (79.2)
Medical Oncology	3 (1.8)
Radiation Oncology	6 (3.6)
Multidisciplinary Clinic	26 (15.5)
Primary Treatment, *n* (%)	
Radical prostatectomy	120 (71.4)
External beam radiotherapy	10 (6)
Hormonal therapy only	19 (11.3)
Active surveillance	11 (6.6)
Untreated	8 (4.8)
Adjuvant Radiation, yes, *n* (%)	23 (13.7)
Salvage Radiation, yes, *n* (%)	11 (6.5)
Hormonal Therapy, yes, *n* (%)	48 (28.6)

*Data available for 158/168 patients

### Internal consistency

Table 2 shows the descriptive statistics and analysis of internal consistency (Cronbach’s α). The EPIC-CP domains scores ranged from 2.4–7.3, with a total mean score of 19.6 ± 10. Cronbach’s α coefficients were as follows for each domain: urinary incontinence (four items) = 0.75, urinary irritation/obstruction (three items) = 0.8, bowel (three items) = 0.78, sexual (three items) = 0.64, and vitality (0.62). The total Cronbach’s α coefficient was 0.84 demonstrating an acceptable internal consistency.

**Table 2.** Descriptive statistics and analysis of internal consistency (Cronbach’s alpha)

	Mean (SD)	Median (25,75th percentiles)	Range	Cronbach’s alpha
Urinary incontinence	3.8 (3.4)	3 (1, 6)	0–12	0.75
Urinary irritation/obstruction	4.0 (3.2)	3 (1, 6)	0–12	0.80
Bowel	2.4 (2.7)	2 (0, 3)	0–12	0.78
Sexual	7.3 (3.4)	8 (5, 10)	0–12	0.64
Vitality	2.5 (2.6)	2 (0, 4)	0–12	0.62
Total	19.6 (10.4)	18 (13, 24)	0–49	0.84

SD = Standard Deviation

### Test–retest reliability

The ICCs quantifying the test–retest reliability are outlined in Table 3. The ICCs of the domain scores ranged from 0.33–0.66. Urinary irritation/obstruction, bowel and sexual domain ICCs were below our defined *a priori* adequate threshold of 0.5. However, the total ICC was acceptable at 0.64.

**Table 3.** Interclass correlation coefficients (ICC) quantifying test-retest reliability

	Mean (standard deviation)		ICC	Standard error
	Time 1	Time 2	(95% confidence interval)	of measurement
Urinary incontinence	3.9 (3.2)	3.4 (3.0)	.66 (.48, .79)	1.8
Urinary irritation/obstruction	3.7 (3.1)	3.3 (2.6)	.42 (.17, .62)	2.2
Bowel	1.9 (2.3)	2.0 (2.6)	.33 (.07, .56)	2.0
Sexual	7.0 (3.0)	7.3 (2.6)	.37 (.11, .58)	2.2
Vitality	2.5 (2.6)	2.5 (2.3)	.55 (.33, .71)	1.7
Total	18.9 (8.4)	18.3 (8.2)	.64 (.44, .78)	5.1

ICC = Intraclass Coefficient Correlations

## Discussion

The overarching design of the present study was to validate the internal consistency and evaluate the test–retest reliability of a PRO measure that had been translated into an Arabic version for patients with PCa. The five domains encompassed: urinary incontinence, urinary irritation/obstruction, bowel, sexual, and vitality/hormonal health functions, and serve to encapsulate a standardised guide for physicians and patients in determining both which PCa treatments are preferred, and which functional outcomes are reasonably found in similar patients before and after treatment. Taking a previously harmonised Arabic version of the EPIC-CP, translated using specifications created by ISPOR, this PRO questionnaire detailing HRQL standards was distributed amongst patients with PCa in four Arabic-speaking countries for a total of 168 patients. Internal consistency and test–retest reliability were created for respective domain scores using Cronbach’s α and ICC analyses. To our knowledge this is the first generated Arabic template of any EPIC version, therefore satisfying a major need in the Arabic community where PCa rates have risen over the previous decades [13–15].

Compared to participants from the original EPIC-CP validation cohort [5], our present sample had a similar median age of participants. The median PSA level was relatively higher, and this could be due to delayed diagnosis due to the absence of PCa screening programmes, as well as specific population-related factors in the Arab world [18]. Similar to the original validation cohort, our present sample included patients mostly with Grade Group 2 and 3 PCa and cT1 stage; however, our sample had many more patients with cTX and if diagnosed, these patients will likely have higher grade groups and PCa stages due to less screening and delayed diagnosis. Recent data from Lebanon, Saudi Arabia and Kuwait revealed high rates of Stage 4 PCa at presentation or pT3 pathology after prostatectomy [13,19,20]. Our present sample was much more likely to be mainly treated by urologists’ vs radiation oncologist, medical oncologist, or in a multidisciplinary clinic; and by radical prostatectomy vs radiation therapy, hormonal therapy only, active surveillance or no treatment compared to the original validation cohort.

The translated Arabic EPIC-CP overall internal consistency was satisfactory with a Cronbach’s α of 0.84. Sexual and vitality/hormonal health function domain scores fell slightly below our *a priori* defined criteria for internal consistency. Both domains had similar coefficients in previously translated versions of EPIC-16 into Chinese, and the vitality/hormonal domain had similar coefficients in EPIC-CP for Portuguese, EPIC-26 for French, Italian, and EPIC-50 for German-speakers [6,8,9,11,21]. These two domains also correlated with scores from the original EPIC-CP cohort [5]. With regards to the low hormonal/vitality scores, which was present in almost all translation studies, Chang et al. [5] attributed this to the broad, systemic nature of this domain. Our overall test–retest ICC was acceptable at 0.64. The relatively low ICCs were expected given the lower number of items in each domain compared to the original EPIC-50 and EPIC-26. Therefore, this Arabic version of EPIC-CP received sufficient internal consistency and test–retest reliability, thereby demonstrating acceptable standards for implementation into clinical use.

Arabic-speaking countries have their own distinct accents, and with it their own cultural and language differences. The present study has tried to mitigate these differences by incorporating patients with PCa from multiple Arabic-speaking countries over varying geographical locations. Thus, our translated version would be applicable to all Arabic-speaking patients. We believe this tool would improve care of immigrants from this area and mitigate the effect of language barrier during clinical encounters. It will also help these patients to enrol in PCa trials in developed countries and foster the PCa research in the Arab world.

The present study has several limitations. The translated EPIC-CP was tested in patients with PCa undergoing a variety of treatment methods. However, as mentioned earlier, given that most of the authors are urologists (with access mainly to urology clinic patients) in our study, the majority of participants were treated by radical prostatectomy, and this varies from the original EPIC-CP cohort. Given that different treatments may lead to different side-effects, it is worthwhile to further test this questionnaire by distributing the Arabic translated EPIC-CP to patient populations undergoing PCa treatment not included in the present study. Nevertheless, EPIC-CP was translated with the specifications given by ISPOR and as such the translation process warrants no threat to validity.

The EPIC-CP represents a highly abbreviated version of the original 50-question EPIC PRO measure for HRQL before and after PCa treatment. The feasibility and completion rate for this 16-question Arabic version represents an opportunity for both a more efficient and more patient-friendly version for in-house clinical usage in modern day industrious practices. The internal consistency and test–retest reliability of the EPIC-CP demonstrates this convenience does not sacrifice overall effectiveness of the survey when translated for Arabic-speaking patients. Going forward, the authors recommend the implementation of this validated Arabic translated EPIC-CP for Arabic speaking patients and following their HRQL before and after PCa treatment.

## Supplementary Material

Supplemental Material

## Abbreviations

EPIC-CP: Expanded Prostate Cancer Index Composite for Clinical Practice; HRQL: Health-related quality of life; ICC: interclass correlation coefficient; IQR: interquartile range; ISPOR: International Society for Pharmacoeconomics and Outcomes Research; PCa: prostate cancer; PROs: patient reported outcomes
