# Supplementary material for: Creation and validation of the harmonized Arabic version of the Expanded Prostate Cancer Index Composite for Clinical Practice (EPIC-CP)
Source: Arab J Urol. 2021 Nov 28;20(2):88–93. doi: 10.1080/2090598X.2021.2002636 (PMC9067989; doi:10.1080/2090598X.2021.2002636)
Supplement: Supplemental Material [file TAJU_A_2002636_SM5610.pdf]

مؤشر سرطان البروستاتا الموسّع والمركب للممارسة السريرية (EPIC-CP)

أداة سريرية لقياس صحة الجهاز البولي والأمعاء والصحة الجنسية والحيويّة والهرمونية التاريخ: ___/___/___

المرضى: يرجى الاجابة على الاسئلة التالية عن طريق تحديد مربع الاختيار المناسب. جميع الاسئلة تدور حول صحتك والأعراض التي تعرضت لها في الاسابيع الاربعة الأخيرة. اختر إجابة واحدة لكل سؤال.

١. بشكل عام، ما حجم مشكلة الوظيفة البولية (عملية التبول) بالنسبة إليك؟

☐ لا مشكلة ☐ مشكلة صغيرة جدا ☐ مشكلة صغيرة ☐ مشكلة متوسطة ☐ مشكلة كبيرة

٢. أي من العبارات التالية تصف التحكم البولي الخاص بك بشكل أفضل؟ ____

٠☐ تحكُّم كلي ١☐ تقطُّر من وقت الى آخر ٢☐ تقطُّر متكرر ٤☐ لا يوجد تحكُّم

٣. ما عدد الفوَط الصحية أو حفاضات البالغين التي تستخدمها يومياً لتسرب البول؟ ____

٠☐ لا أستخدم ١☐ حفاضة واحدة يومياً ٢☐ حفاضتان يومياً ٤☐ ثلاثة حفاضات أو أكثر يوميا

٤. ما حجم المشكلة التي تَسبَّب فيها تقطُّر وتسُّرب البول، إن وُجدت؟ ____

٠☐ لا مشكلة ١☐ مشكلة صغيرة جدًا ٢☐ مشكلة صغيرة ٣☐ مشكلة متوسطة ٤☐ مشكلة كبيرة

الطاقم الطبي: أضف الإجابات من الأسئلة ٢-٤ لحساب درجة اعراض سلس البول (من أصل ١٢): ☐

٥. ما حجم المشكلة بالنسبة لكل من الأعراض التالية، إن وُجدت:

	لا توجد مشكلة	مشكلة صغيرة جدا	مشكلة صغيرة	مشكلة متوسطة	مشكلة كبيرة	
أ. ألم أو حرقان أثناء التبول	☐ ٠	☐ ١	☐ ٢	☐ ٣	☐ ٤	____
ب. ضعف تدفُّق البول / عدم إفراغ المثانة كلياً	☐ ٠	☐ ١	☐ ٢	☐ ٣	☐ ٤	____
ج. الحاجة المتكررة للبول	☐ ٠	☐ ١	☐ ٢	☐ ٣	☐ ٤	____

الطاقم الطبي: أضف الإجابات من الأسئلة ٥أ-٥ج لحساب درجة التهيج أو الانسداد البولي (من أصل ١٢): ☐

٦. ما حجم المشكلة بالنسبة لكل من الأعراض التالية، إن وُجدت:

	لا توجد مشكلة	مشكلة صغيرة جدا	مشكلة صغيرة	مشكلة متوسطة	مشكلة كبيرة	
أ. ألم في الشرج أو حاجة مُلحّة للتبرّز	☐ ٠	☐ ١	☐ ٢	☐ ٣	☐ ٤	____
ب. زيادة تكرار التبرّز	☐ ٠	☐ ١	☐ ٢	☐ ٣	☐ ٤	____
ج. مشاكل التبرّز بشكل عام	☐ ٠	☐ ١	☐ ٢	☐ ٣	☐ ٤	____

الطاقم الطبي: أضف الإجابات من الأسئلة ٦أ-٦ج لحساب درجة أعراض الأمعاء (من أصل ١٢): ☐

٧. كيف تقيّم قدرتك على الوصول الى النشوة الجنسية (الذروة)؟ ____

٠☐ جيدة جدا ١☐ جيدة ٢☐ مقبولة ٣☐ ضعيفة ٤☐ ضعيفة جدا أو منعدمة

٨. كيف تصف جودة الانتصاب المعتادة؟ ____

٠☐ انتصاب صَلب بما يكفي للجماع ١☐ انتصاب كافي للإستمناء والمداعبة فقط ٢☐ انتصاب غير كافي لأي نشاط جنسي ٤☐ انتصاب غير كافي لأي نشاط جنسي

٩. بشكل عام، ما مدى المشكلة الناتجة عن الوظيفة الجنسية أو انعدام وجودها عليك؟ ____

٠☐ لا مشكلة ١☐ مشكلة صغيرة جدا ٢☐ مشكلة صغيرة ٣☐ مشكلة متوسطة ٤☐ مشكلة كبيرة

الطاقم الطبي: أضف الإجابات من الأسئلة ٦أ-٦ج لحساب درجة أعراض الأمعاء (من أصل ١٢): ☐

١٠. ما حجم المشكلة بالنسبة لكل من الأعراض التالية، إن وُجدت:

	لا توجد مشكلة	مشكلة صغيرة جدا	مشكلة صغيرة	مشكلة متوسطة	مشكلة كبيرة	
أ. هَبّات ساخنة، آلام أو تضخم الثدي	☐ ٠	☐ ١	☐ ٢	☐ ٣	☐ ٤	____
ب. الشعور بالاكتئاب	☐ ٠	☐ ١	☐ ٢	☐ ٣	☐ ٤	____
ج. نقص الطاقة	☐ ٠	☐ ١	☐ ٢	☐ ٣	☐ ٤	____

الطاقم الطبي: أضف الإجابات من الأسئلة ١٠أ-١٠ج لحساب درجة الأعراض الحيوية/الهرمونية (من أصل ١٢): ☐

الطاقم الطبي: أضف نتائج ملخص الحقول الخمسة لحساب مجموع درجات جودة الحياة لسرطان البروستاتا (من أصل ٦٠): ☐
